# Isotope effects in self-organization of internal transport barrier and concomitant edge confinement degradation in steady-state LHD plasmas

**DOI:** 10.1038/s41598-019-52271-w

**Published:** 2019-11-04

**Authors:** T. Kobayashi, H. Takahashi, K. Nagaoka, M. Sasaki, M. Nakata, M. Yokoyama, R. Seki, M. Yoshinuma, K. Ida

**Affiliations:** 1 National Institute for Fusion Science, National Institutes of Natural Sciences, Toki, 509-5292 Japan; 2 SOKENDAI (The Graduate University for Advanced Studies), Toki, 509-5292 Japan; 3 Research Institute for Applied Mechanics, Kyushu University, Kasuga, 816-8580 Japan

**Keywords:** Magnetically confined plasmas, Phase transitions and critical phenomena

## Abstract

The isotope effect, which has been a long-standing mystery in the turbulent magnetically confined plasmas, is the phenomena that the plasma generated with heavier hydrogen isotope show a mitigated transport. This is on the contrary to what is predicted with the simple scaling theory, in which the heavier ions easily diffuse because of its larger gyro-radius. Thanks to the newly developed analysis method and a comprehensive parameter scan experiment in the steady-state plasmas in the Large Helical Device (LHD), the isotope effect was clearly observed in the self-organized internal transport barrier (ITB) structure for the first time. Comparing the ITB intensity in deuterium (D) and hydrogen (H) plasmas, two distinct hydrogen isotope effects are found: stronger ITB is formed in D plasmas and a significant edge confinement degradation accompanied by the ITB formation emerges in H plasmas. This observation sheds light on a new aspect of the turbulent plasmas regarding how the basic properties of the fluid material affect the turbulent structure formation in the open-system.

## Introduction

Transport in turbulent fluid is known to be highly nonlinear, therefore transition among different states of transport often occurs^1^. A prototypical example is the confinement improvement events in magnetically confined torus fusion plasmas, which sometimes involve the self-regulation of the turbulence structure. Understanding of the structure formation mechanism in such an open-system is one of the most important issues in the modern physics. Since there are multiple controllable parameters and precise diagnostics, the laboratory plasma is an ideal object for attacking this challenging issue. There are two types of confinement transition events: formations of the edge transport barrier (ETB)^2^ and the internal transport barrier (ITB)^3,4^. To reveal the nature of the bifurcation boundary between the bad confinement state, the so-called L-mode, and the improved confinement state, diagrams showing in which operation regimes the transition occurs need to be created. In the case of the ETB, there are several well-defined criteria, i.e., a sharp drop of the edge recycling emission, the pedestal profile formation at the edge, and others^2^. Thanks to them, the diagram of the transition condition is routinely reproduced^5–7^. In contrast, no commonly used definition of the ITB exists so far, although some practical approaches, i.e., the major radius divided by the temperature gradient length exceeding a critical value or a sharply suppressed diffusion coefficient at some specific regimes, are used to identify the ITB structure^3,8–13^. This diversity of the definition makes comprehensive understanding of the bifurcation nature difficult in the case of the ITB.

Another long-standing open issue in the fusion community is the hydrogen isotope effect^14^; the heavier the fuel particles are the better the confinement becomes, contrary to the simple scaling model, the so-called gyro-Bohm scaling. In particular, the hydrogen isotope effect is more obvious in properties of the ETB. For example, the threshold power above which the ETB formation occurs is lowered by a factor with the heavier hydrogen isotope fuel^5–7^. In contrast, isotope effect in ITB has not been systematically discussed to date.

Here, we utilize a newly defined unique parameter that represents the ITB intensity, the so-called profile gain factor^15^, and reveal how the steady-state ITB intensity depends on operation parameters. The gradual transition to the ITB occurs when the density of the plasma is decreased. The ITB is more prominent in deuterium plasmas than in hydrogen plasmas, in particular in the inward shifted magnetic configuration. A significant edge confinement degradation accompanied by the ITB formation emerges in hydrogen plasmas. By the combination of these two isotope effects, higher ion temperature plasmas are realized in deuterium plasmas. Different saturation mechanisms of the ion temperature profile, which may have an isotope dependence, are found in the L-mode and the ITB regime.

## Results

### Experimental set-up

The experiments were conducted in the Large Helical Device (LHD). The plasmas in LHD are stable and quiescent, which are the ideal environment for investigating the basic physics of the ITB. In order to explore parameter dependence of the properties of the ITB, the line averaged density ${\bar{n}}_{{\rm{e}}}$ is scanned in the shot-to-shot basis for three different magnetic configurations with the vacuum magnetic axis positions of ${R}_{{\rm{ax}}}=3.55\,{\rm{m}}$, 3.58 m, and 3.60 m^16^. As the magnetic axis is shifted inward, the magneto-hydro-dynamics (MHD) mode stability deteriorates but the neoclassical properties are optimized^17^. It is empirically known that the strong ITB is formed in the inward shifted configuration and the low density condition^16^. The magnetic field strength is 2.85 T at the magnetic axis in the case of ${R}_{{\rm{ax}}}=3.60\,{\rm{m}}$, and increases by a few percent as ${R}_{{\rm{ax}}}$ is shifted inward. Equivalent datasets are obtained from deuterium (D) and hydrogen (H) plasmas, which are sustained by five neutral beams (NBs). The source gas of the NBs is chosen corresponding to the fuel gas. The lifetime of the ITB is merely determined by the pulse length of the NBs, accompanying no rapid transient behavior nor any major collapse events. The ion temperature ${T}_{{\rm{i}}}$ is measured by the charge exchange recombination spectroscopy^18^, and the electron temperature ${T}_{{\rm{e}}}$ and the electron density ${n}_{{\rm{e}}}$ are given by the Thomson scattering system^19^. The NB absorption profile is calculated by the FIT3D code^20^.

### Isotope effects in ITB

**Figure 1:**
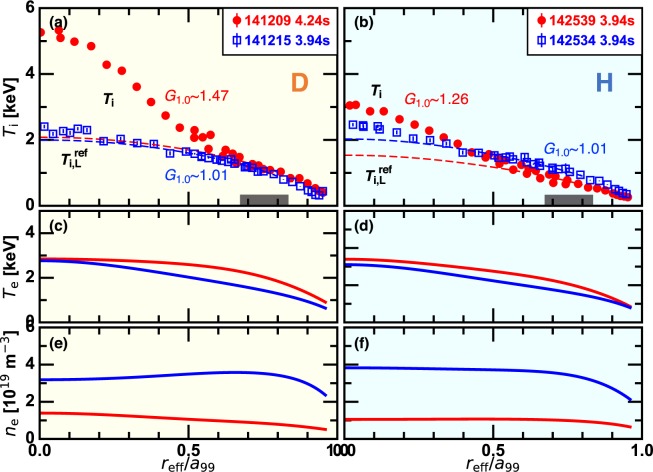
(left) D plasmas and (right) H plasmas. Radial profiles of (**a**,**b**) the ion temperature (**c**,**d**) the electron temperature, and (**e**,**f**) the electron density for the low density discharges (red) and the high density discharges (blue). The values of the profile gain factor are shown in each case in (**a**,**b**). The black rectangles in (**a**,**b**) represent the radial range in which the reference L-mode profile is determined (see text).

Figure 1 shows examples of the measured profiles for D plasmas (left column) and H plasmas (right column), which are chosen as equivalent pairs in terms of ${\bar{n}}_{{\rm{e}}}$. The horizontal axis ${r}_{{\rm{eff}}}/{a}_{99}$ is the effective minor radius normalized by the averaged minor radius in which 99% of the electron kinetic energy is confined. The vacuum magnetic axis position is ${R}_{{\rm{ax}}}=3.55\,{\rm{m}}$ and the low density case (red) and the high density case (blue) are compared. In this article, the figures for D and H plasmas are shown with light yellow panels and light blue panels, respectively, for the sake of clarity. As shown in Fig. 1(a,b), particularly stronger ITB is formed in the low density D plasma. The electron temperature is slightly higher in H plasmas than in D plasmas as shown in Fig. 1(c,d) because of the larger fraction of the tangential NB power, which is mainly absorbed by electrons.

The ITB intensity is defined by the profile gain factor^15^. See Methods section for definition. Dashed curves in Fig. 1(a,b) correspond to the reference L-mode profiles, ${T}_{{\rm{i}},{\rm{L}}}^{{\rm{ref}}}$, which are the profiles synthesized to satisfy the empirical confinement scaling of the diffusion coefficient in the L-mode, $\chi \propto {T}_{{\rm{i}}}$. In the high density discharges, the *T*_i_ profile is well reproduced by the ${T}_{{\rm{i}},{\rm{L}}}^{{\rm{ref}}}$ profile, indicating that the diffusion coefficient scales as the L-mode manner. In contrast, the ${T}_{{\rm{i}}}$ profile considerably surpasses the ${T}_{{\rm{i}},{\rm{L}}}^{{\rm{ref}}}$ profile in ${r}_{{\rm{eff}}}/{a}_{99} < 0.6$ in the low density discharges, being regarded as the ITB formation. The profile gain factor ${G}_{1.0}$ is defined as the ratio of the ion kinetic energy calculated with ${T}_{{\rm{i}}}$ to that with ${T}_{{\rm{i}},{\rm{L}}}^{{\rm{ref}}}$. In the high density discharges ${G}_{1.0}$ is approximately the unity. The low density discharges have ${G}_{1.0}$ larger than the unity, and ${G}_{1.0}\sim 1.47$ in the D plasma is meaningfully larger than ${G}_{1.0}\sim 1.26$ in the H plasma. Only in the H plasmas an edge confinement degradation accompanied by the ITB formation occurs, which results in only the marginal increase of the core ion temperature even with the ITB. This tendency is general in H plasmas in a wide parameter range, which is shown below.

**Figure 2:**
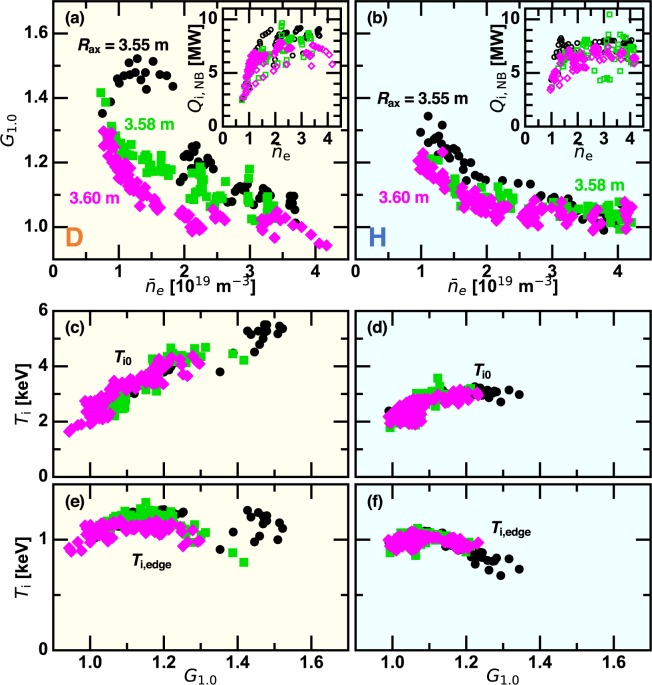
(left) D plasmas and (right) H plasmas. (**a**,**b**) The line averaged density dependence of the profile gain factor, (**c**,**d**) the central ion temperature, and (**e**,**f**) the edge ion temperature plotted against the profile gain factor. (insert) The line averaged density dependence of the absorption power of NBs by ions.

Figure 2(a,b) show ${G}_{1.0}$ versus ${\bar{n}}_{{\rm{e}}}$ in D and H plasmas, respectively. The dataset is established by 42 shots for D plasmas and 43 shots for H plasmas. Eight frames of the ${T}_{{\rm{i}}}$ profile are obtained in one discharge, which are regarded as independent data. The NB power absorbed by ions ${Q}_{{\rm{i}},{\rm{NB}}}$ is plotted as a function of ${\bar{n}}_{{\rm{e}}}$ in inserts of Fig. 2(a,b), showing decaying ${Q}_{{\rm{i}},{\rm{NB}}}$ with ${\bar{n}}_{{\rm{e}}}$ in ${\bar{n}}_{{\rm{e}}} < 1.5\times {10}^{19}\,{{\rm{m}}}^{-3}$ because of the larger shine-through. Approximately 10% higher ${Q}_{{\rm{i}},{\rm{NB}}}$ in D plasmas than in H plasmas is due to the higher perpendicular NB power. When ${\bar{n}}_{{\rm{e}}}$ is relatively high, the plasma is in the L-mode as shown by the profile gain factor of ${G}_{1.0} < 1.1$. As ${\bar{n}}_{{\rm{e}}}$ is decreased, ${G}_{1.0}$ increases nonlinearly. When ${R}_{{\rm{ax}}}$ is shifted inward, ${G}_{1.0}$ tends to increase. In the case of ${R}_{{\rm{ax}}}=3.55\,{\rm{m}}$, the stronger ITB with larger ${G}_{1.0}$ is formed in D plasmas rather than in H plasmas, which is the apparent isotope difference. This tendency becomes less clear as ${R}_{{\rm{ax}}}$ is shifted outward. In a different dataset with ${R}_{{\rm{ax}}}=3.60\,{\rm{m}}$, stronger ITBs in D plasmas were found too^21^.

We discuss how the central ion temperature ${T}_{{\rm{i}}0}$ and the edge ion temperature ${T}_{{\rm{i}},{\rm{edge}}}$ relate to the ITB intensity. These are defined by the averaged value of ${T}_{{\rm{i}}}$ in $0.07 < {r}_{{\rm{eff}}}/{a}_{99} < 0.15$ and $0.68 < {r}_{{\rm{eff}}}/{a}_{99} < 0.83$, respectively. Note that ${T}_{{\rm{i}},{\rm{edge}}}$ reflects the confinement capability at the edge, while ${T}_{{\rm{i}}0}$ is determined both by the edge confinement and the ITB intensity. In D plasmas, ${T}_{{\rm{i}}0}$ is almost proportional to ${G}_{1.0}$, while ${T}_{{\rm{i}},{\rm{edge}}}$ is insensitive to ${G}_{1.0}$. On the contrary, ${T}_{{\rm{i}}0}$ saturates in ${G}_{1.0} > 1.1$ in H plasmas because the edge confinement degrades as the core confinement is improved as shown by decreasing ${T}_{{\rm{i}},{\rm{edge}}}$ with ${G}_{1.0}$. The ITB-concomitant edge confinement degradation clearly seen in H plasmas is another isotope effect.

### Local variables that contribute to the isotope effects

**Figure 3:**
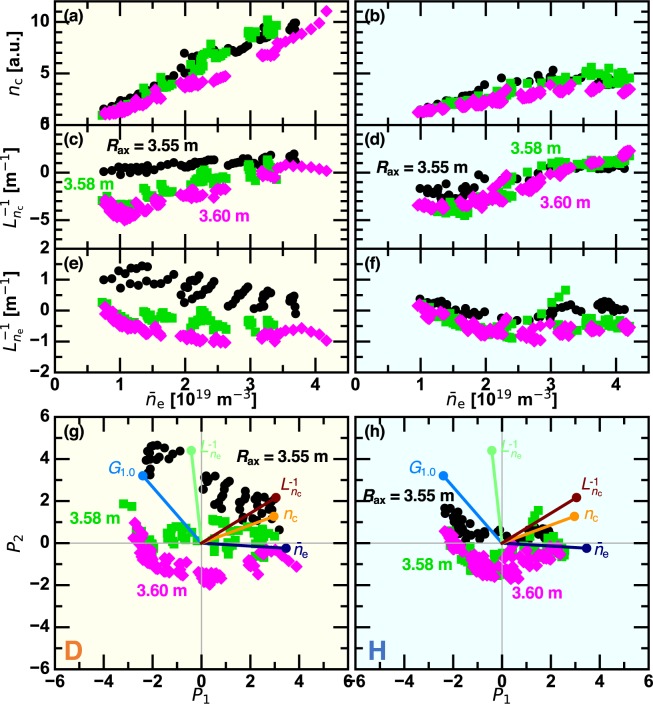
(left) D plasmas and (right) H plasmas. The line averaged density dependence of (**a**,**b**) the carbon impurity density, (**c**,**d**) the inverse gradient length of the carbon impurity density, and (**e**,**f**) the inverse gradient length of the electron density at ${r}_{{\rm{eff}}}/{a}_{99}=0.6$. (**g**,**h**) Scatter plot of the principal component score and eigen vectors of the original variables.

Local variables that play a role on the ITB properties and vary with ${\bar{n}}_{{\rm{e}}}$ are investigated. Figure 3(a–f) show ${\bar{n}}_{{\rm{e}}}$ dependence of the carbon impurity density ${n}_{{\rm{c}}}$ and the inverse gradient lengths of ${n}_{{\rm{c}}}$ and ${n}_{{\rm{e}}}$, denoted as ${L}_{{n}_{{\rm{c}}}}^{-1}$ and ${L}_{{n}_{{\rm{e}}}}^{-1}$, respectively, at ${r}_{{\rm{eff}}}/{a}_{99}=0.6$. Here, the inverse gradient length of an arbitrary variable $\Psi$ is defined as ${L}_{\Psi }^{-1}=-\,{\Psi }^{-1}\partial \Psi /\partial {r}_{{\rm{eff}}}$, where the gradient is estimated by the linear regression for the data points in $\pm 0.1\times {a}_{99}$ of the radius of interest. Therefore, the positive value of the inverse gradient length corresponds to the density peaking. Interrelation among the five-dimensional data points in $({\bar{n}}_{{\rm{e}}},{G}_{1.0},{n}_{{\rm{c}}},{L}_{{n}_{{\rm{c}}}}^{-1},{L}_{{n}_{{\rm{e}}}}^{-1})$ is resolved by the principal component analysis (PCA). Approximately 90% of the information in the dataset can be expressed in the two-dimensional space of the first and second principal components, ${P}_{1}$ and ${P}_{2}$, whose cumulative contribution ratios are 0.51 and 0.891, respectively. Figure 3(g,h) show the results of the PCA. In the diagrams, symbols correspond to the principal component score, showing how the data points depend on ${P}_{1}$ and ${P}_{2}$. The relations with the five variables are reflected by the eigen vectors displayed as the lines ended with a circle. The angle between a pair of vectors indicates how strongly these vectors depend upon each other. In order to quantify the correlation between the ITB intensity and other variables, cosine of the angles between the eigen vector of ${G}_{1.0}$ and other eigen vectors are listed in Table 1. In addition to the anti-correlation between ${G}_{1.0}$ and ${\bar{n}}_{{\rm{e}}}$, there is a considerable correlation between ${G}_{1.0}$ and ${L}_{{n}_{{\rm{e}}}}^{-1}$, implying a possible impact of the density peaking on the ITB formation^22^. Unlike the ITB discharge with the assistance of the carbon impurity pellet^23,24^, ${n}_{{\rm{c}}}$ and ${L}_{{n}_{{\rm{c}}}}^{-1}$ provide less impact on the ITB intensity. The vertical expansion of the points in different ${R}_{{\rm{ax}}}$ conditions is larger in D plasmas than in H plasmas, showing that ${R}_{{\rm{ax}}}$ dependence of ${G}_{1.0}$ and ${L}_{{n}_{{\rm{e}}}}^{-1}$ is more significant in D plasmas.

**Table 1:** Cosine of angle of eigen vectors with respect to *G*_1.0_ in the principal component analysis.

${\bar{{\boldsymbol{n}}}}_{{\bf{e}}}$	*G*_1.0_	*n*_c_	${{\boldsymbol{L}}}_{{{\boldsymbol{n}}}_{{\bf{c}}}}^{-{\bf{1}}}$	${{\boldsymbol{L}}}_{{{\boldsymbol{n}}}_{{\bf{e}}}}^{-{\bf{1}}}$
−0.654	1	−0.235	−0.025	0.853

## Discussion

**Figure 4:**
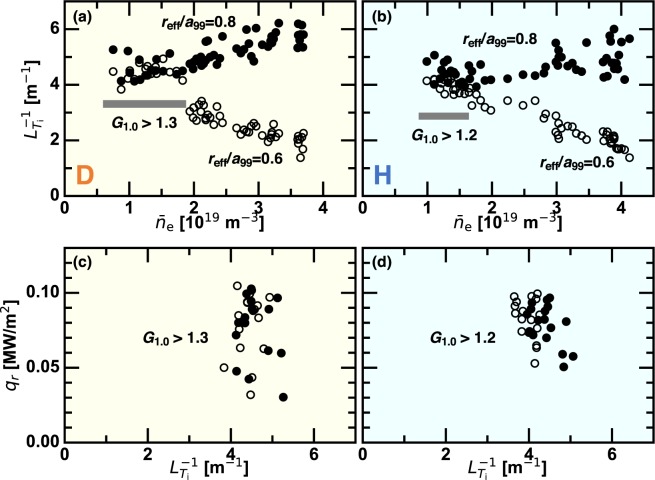
(left) D plasmas and (right) H plasmas. (**a**,**b**) The line averaged density dependence of the inverse ion temperature gradient length at ${r}_{{\rm{e}}ff}/{a}_{99}\mathrm{=0.6}$ and ${r}_{{\rm{eff}}}/{a}_{99}=0.8$ and (**c**,**d**) diagram of ion heat flux versus inverse ion temperature gradient length when the ITB is formed.

When the ITB is formed, the scaling law $\chi \propto {T}_{{\rm{i}}}$ is violated in the core region. This fact anticipates the different profile saturation mechanisms existing in the L-mode regime and the ITB regime. In order to investigate how the profile shape is restricted, the inverse ion temperature gradient length ${L}_{{T}_{{\rm{i}}}}^{-1}$ is plotted as a function of ${\bar{n}}_{{\rm{e}}}$ in Fig. 4(a,b). As a representative example, the case of ${R}_{{\rm{ax}}}=3.55\,{\rm{m}}$ is shown. The strong ITB regimes, ${G}_{1.0} > 1.3$ in D plasmas and ${G}_{1.0} > 1.2$ in H plasmas, are indicated by the gray rectangles. In the high density L-mode regime ${L}_{{T}_{{\rm{i}}}}^{-1}$ at ${r}_{{\rm{eff}}}/{a}_{99}=0.8$ is much larger than ${L}_{{T}_{{\rm{i}}}}^{-1}$ at ${r}_{{\rm{eff}}}/{a}_{99}=0.6$, while they overlap when the ITB is formed. Constant ${L}_{{T}_{{\rm{i}}}}^{-1}$ over a range of the radius in the ITB regime implies the profile stiffness^25^ emerging as the profile saturation mechanism. This tendency is seen in other ${R}_{{\rm{ax}}}$ cases, too, although it is less clear, because the ITB is relatively weak with outward shifted ${R}_{{\rm{ax}}}$. It is essential to examine robustness of ${L}_{{T}_{{\rm{i}}}}^{-1}$ against the local heat flux ${q}_{r}$ to judge whether the profile stiffness occurs. Scan in ${q}_{r}$ is performed by use of the slowly raising phase of the NB absorption in the low density regime, where the slowing-down time is relatively long. Figure 4(c,d) show the diagram of ${q}_{r}$ versus ${L}_{{T}_{{\rm{i}}}}^{-1}$ for the ITB regimes. Constant ${L}_{{T}_{{\rm{i}}}}^{-1}$ against varying ${q}_{r}$ evidences emergence of the profile stiffness. In the critical gradient theory^26^, it is predicted that the ion temperature gradient (ITG) driven turbulence is excited when the profile stiffness emerges. Indeed, the ion scale turbulence propagating in the ion diamagnetic drift direction has been observed when the ITB is formed^27^, which is regarded as the linearly unstable ITG turbulence^26^. In contrast, in the L-mode regime ${L}_{{T}_{{\rm{i}}}}^{-1}$ is not constant in radius but increases with radius, implying that the profile is not fully regulated by the ITG turbulence. The saturation mechanism of the ion temperature profile in the L-mode is an interesting subject for future investigation. The different saturation mechanisms of the ion temperature profile possibly imply that different turbulences play a role in the L-mode and the ITB regime, which have an isotope dependence.

## Summary

In conclusion, new aspects of the isotope effect in the ITB formation were found in LHD: the stronger ITB in D plasmas and the ITB-concomitant edge confinement degradation in H plasmas. In the L-mode, the ion temperature profile was characterized by the dome-shaped profile, in which the diffusion coefficient linearly scales with the local ion temperature, while it was restricted by the profile stiffness in the ITB regime. The different saturation mechanisms of the ion temperature profile possibly imply that different turbulences play roles in the L-mode and the ITB regime, which have an isotope dependence. This point can be a key to unveil the different confinement properties in tokamaks and stellarator/heliotrons, and is an interesting subject for future investigations.

## Methods

### Large Helical Device

LHD is a magnetically confined fusion plasma device of the heliotron magnetic configuration. The representative major and minor radii of the torus plasma are 3.6 m and ~0.6 m, respectively. The confinement magnetic field is mainly produced by the external helical coils, therefore the plasma current need not be maintained. In the vacuum magnetic field configuration, the rotational transform, $\iota /2\pi =1/q$, where *q* is the safety factor, monotonically increases with the radius. The rational surfaces of $\iota /2\pi =0.5$ and 1 typically exist at the core and the edge, respectively. For the comparison study of deuterium (D) and hydrogen (H) plasmas, baking and glow discharge cleaning were performed before the experiments, which significantly contribute to increase the D/H purity. The plasma is heated by five neutral beams (NBs); three are tangentially injected and two are perpendicularly injected. The total port-through power is ~20 MW. Heating power ratio of the tangentially injected NBs to the perpendicularly injected NBs are 6 MW/14 MW and 11 MW/9 MW in D and H plasmas, respectively. Because of this difference, the absorption power by ions is slightly higher in D plasmas.

### Profile gain factor

The ITB intensity is defined by the profile gain factor^15^. In tokamaks, the profile stiffness is routinely observed in non-ITB plasmas, in which the inverse ion temperature gradient length ${L}_{{T}_{{\rm{i}}}}^{-1}$ is constant over a wide radial range and is robust for increasing heating power^25^. When the ITB is formed, the profile stiffness is violated so that ${L}_{{T}_{{\rm{i}}}}^{-1}$ surpasses its L-mode value, which is a reasonable criterion for the ITB^3,11–13^. In contrast, the L-mode plasmas in LHD are characterized not by the profile stiffness^28^ but by the dome-shaped ion temperature profile, where the confinement degrades and the gradient is lowered as the ion temperature increases towards the core^29^. Therefore, the definition of the ITB used in tokamaks is not appropriate for LHD. We define a unique scalar parameter, the profile gain factor, as a new criterion of the ITB intensity. The profile gain factor quantifies degree of the confinement improvement with respect to the reference L-mode profile ${T}_{{\rm{i}},{\rm{L}}}^{{\rm{ref}}}$, which has the typical L-mode scaling. The solution of the steady-state energy conservation equation with the diffusion coefficient $\chi =k{T}_{{\rm{i}}}^{\alpha }$, where *α* and *k* are the exponent factor and the proportionality factor, respectively, is used as ${T}_{{\rm{i}},{\rm{L}}}^{{\rm{ref}}}$. From the $\chi$ versus ${T}_{{\rm{i}}}$ diagram in the typical L-mode plasmas in LHD, $\alpha =1$ is found to approximately hold^15^ and is fixed for the analysis in this article. Note that in the global scaling in LHD, $\alpha =1.5$ fits a large database^30^, which is consistent with the gyro-Bohm scaling. The factor *k* is determined using the edge profile data, in which the confinement behaves as the L-mode even when the ITB is formed in the core^29^. The factor *k* is evaluated in $0.68 < {r}_{{\rm{eff}}}/{a}_{99} < 0.83$ that is shown as black rectangles in Fig. 1(a,b), where the result is insensitive to the choice of this specific range. The profile gain factor ${G}_{1.0}$ is defined as the ratio of the ion kinetic energy calculated with ${T}_{{\rm{i}}}$ to that with ${T}_{{\rm{i}},{\rm{L}}}^{{\rm{ref}}}$, i.e.,

1 $${G}_{1.0}=\frac{\int \,nT(r)dV}{\int \,n{T}_{{\rm{L}}}^{{\rm{ref}}}(r)dV},$$

where $\int \,dV$ represent the torus volume integral. Here, the subscript 1.0 denotes the employed value of the *α* factor.

### Principal Component Analysis

In fusion experiment, it is generally difficult to vary a single parameter independently. In case the multiple parameters change simultaneously, the principal component analysis is useful to decompose their relations. New coordinates called the principal components are defined to emphasize variation of the data so that the projection of the original data and original coordinate vectors on the new coordinates provides a better interpretation. Cumulative contribution ratios are defined as a function of the number of the principal components, showing how much ratio of the original data information is expressed by the set of the principal components. When the cumulative contribution ratio of the second principal component is high enough, the data can be better visualized by the two-dimensional principal component’s space.
